# Establishment of rat allogenic vein replacement model and pathological characteristics of the replaced vessels

**DOI:** 10.3389/fsurg.2022.984959

**Published:** 2022-09-09

**Authors:** Zhangyong Ren, Songping Cui, Shaocheng Lyu, Jing Wang, Lin Zhou, Yanan Jia, Qiang He, Ren Lang

**Affiliations:** Department of Hepatobiliary and Pancreaticosplenic Surgery, Beijing Chaoyang Hospital, Capital Medical University, Beijing, China

**Keywords:** vein transplantation, intimal hyperplasia, animal model, vein replacement, vascular stenosis

## Abstract

**Background:**

With the advancement of vascular anastomosis techniques in recent years, radical surgery for tumors combined with venous vascular resection and reconstruction has been widely used. This study intends to establish two different rat vein replacement models, and further analyze the pathological changes of blood vessels after replacement.

**Methods:**

Brown-Norway (BN) rats were selected as donors and recipients, randomly divided into control group, cuff group (1-week group, 2-week group, and 4-week group), and suture group (1-week group, 2-week group, and 4-week group), with 6 rats in each group. The perioperative conditions, inner diameter, flow velocity and histopathological changes of the replaced vessels at different time points were analyzed.

**Results:**

Both cuff group and suture group can safely establish the rat vein replacement model. From the surgical operation, the operation time and venous cross-clamp time in the cuff group were shorter than those in the suture group (*P* < 0.05). At 2 and 4 weeks after operation, the diameter of suture group was wider than that of cuff group, and the flow rate was faster (*P* < 0.05). With prolonged postoperative survival, the wall of the replaced vessels underwent infiltration of CD4+ and CD8+ lymphocytes and high TGF-β1 gene expression. This leads to the proliferation of blood vessels and intimal layer. The results of vascular pathological staining showed that the infiltration degree of CD4+ lymphocytes at 2 weeks after operation and CD8+ lymphocytes at 4 weeks after operation in the suture group was lighter than that in the cuff group (*P* < 0.05). Meanwhile, TGF-β1 gene content at 4 weeks after operation in suture group was significantly lower than that in cuff group (*P* < 0.05).

**Conclusion:**

Compared with cuff method, suture method is more suitable for the study of long-term pathological changes after vein replacement in rats. The main pathological changes in the long term after venous replacement in syngeneic background may be vascular fibrosis caused by inflammatory cell infiltration.

## 1. Introduction

With the progress of vascular anastomosis technology and the update of the concept of comprehensive treatment of tumors in recent years, more and more attention has been paid to radical surgery of tumors combined with venous vascular resection and reconstruction (1–4). For vascular replacement materials that can be used for reconstruction, there are currently clinical reports of autologous veins, autologous parietal peritoneum, autologous falciform ligament, artificial blood vessels and allogeneic blood vessels (5–7). Autologous vessels are less used due to caliber matching and surgical trauma, and for artificial blood vessels, the long-term patency rate is suboptimal. In contrast, allogeneic blood vessels have the advantages of rich sources, easy matching of caliber, and good histocompatibility, which are ideal vein replacement materials in clinical work (8–10).

However, the long-term after allogeneic vascular replacement may lead to thrombosis of the replaced vessel wall, intimal scar hyperplasia, vascular rejection and other phenomena due to local specific and non-specific inflammatory effects (11–13), and ultimately cause vascular stenosis, affecting the long-term prognosis of patients. Therefore, it is important to study the long-term pathological changes of the replaced vessels after allogeneic vein replacement for the prevention and treatment of vascular stenosis. However, it is difficult to obtain pathological specimens after allogeneic vascular replacement in clinical practice, and there are relatively few reports on small animal models of vein transplantation. Therefore, two different rat models of vein replacement were constructed by “cuff method” and “suture method”, and the pathological changes of the replaced vessels after surgery were further analyzed and compared between the two methods.

## 2. Materials and methods

### 2.1. Experimental animal

Specific pathogen free (SPF) grade male Brown-Norway (BN) rats aged 6–8 weeks and weighing about 200 g were selected. The experimental animals were purchased in Beijing Weitong Lihua Animal Experimental Co., Ltd. and were raised in the Medical Research Center of Beijing Chaoyang Hospital to maintain normal room temperature, clean environment, feed ordinary feed and ensure the circadian rhythm of rats [12-h light:12-h dark (12l:12D)].

### 2.2. Perioperative management

All rats were fasted and deprived of water for 6 h before surgery and received 1% isoflurane inhalation for surgical anesthesia. Following surgery, rats were placed in a 37°C warming stage for anesthesia recovery. All rats also required fasting and water deprivation for 12 h after surgery, and returned to normal feeding after 12 h. At the time of specimen acquisition, we opened the abdominal cavity of the rat to obtain tissue and subsequently sacrificed it by exsanguination from the inferior vena cava. All operations are in line with the ethical principles of laboratory animal welfare and approved by the Ethics Committee of Beijing Chaoyang Hospital (No. 2020-D-332).

### 2.3. Surgical procedures

#### 2.3.1. Experimental grouping

The experiment was grouped using the random seed number method and randomly divided into seven groups of six rats each, for a total of 42 BN rats. They were divided into control group, cuff group (1-week group, 2-week group and 4-week group) and suture group (1-week group, 2-week group and 4-week group). For 1-week group, 2-week group and 4-week group, rats will be sacrificed at corresponding time points to obtain specimens for further analysis. Another 36 BN rats were used as vein donors for surgical procedures. The surgical operation was completed according to the experimental groups, and the rats were sacrificed according to different time points to obtain samples for subsequent analysis and detection.

#### 2.3.2. Cuff replacement model

##### 2.3.2.1. Donor vein acquisition

**Figure 1:**
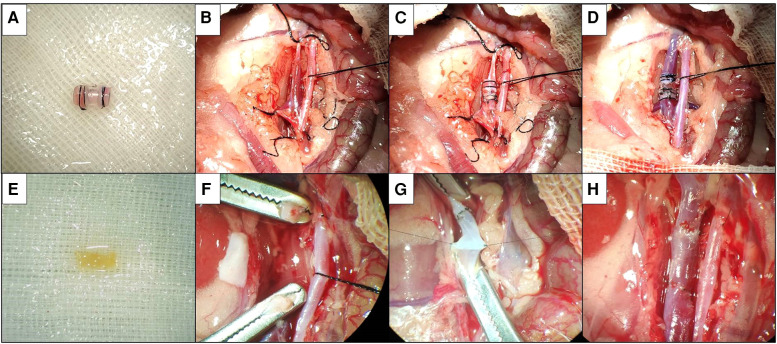
Two kinds of vein replacement model. (**A**) Donor venous cuff by cuff method. (**B**) Silk suture blocks distal and proximal veins. (**C**) Donor venous cuff is implanted after about 2/3 of the circumference of anterior wall of recipient vein is cut. (**D**) Silk suture fixes venous cuff to open venous blood flow. (**E**) Suture method of donor venous segment. (**F**) Dissection after vascular clamp blocks distal and proximal veins. (**G**) Continuous suture after suspension of venous wall. (**H**) After the completion of suture, open venous blood flow.

After skin preparation and disinfection of the abdomen of donor rats, a 2-cm-long incision was made below the xiphoid process into the abdomen, and the subhepatic vein was exposed after separation of the bowel. After separating the vein from the surrounding tissue space, the vein was exposed throughout, and the distal and proximal veins were blocked, respectively, to obtain a vein segment about 7 mm in length, washed with heparinized saline and placed in heparinized saline at 4°C. The removed vein segment was exited from the inside of a thin-walled polyethylene plastic tube 5 mm in length, and both ends were reflexed and sleeved on the plastic tube to form a venous cuff (Figure 1A), which was used as the donor vessel for the cuff method.

##### 2.3.2.2. Donor vein placement

The preparation of recipient rats and the mobilization of veins were the same as before. Temporarily block the vein at the distal and proximal ends of the vein with vascular clips or silk sutures (Figure 1B), cut an incision of about 2/3 circumference in the middle of the anterior wall of the free segment vein, insert one end of the donor vein cuff into the recipient vein, then place the other end of the venous cuff into the recipient vein, completely place the heparinized saline to discharge the air in the lumen of the venous cuff and the recipient vein, adjust the donor vein to the appropriate position and then fix it with 6–0 silk suture (Figure 1C). The posterior 1/3 wall of the recipient vein was divided, and the distal and proximal ends of the vein were blocked in turn (Figure 1D). If there is no active bleeding and the vessel is patent, wash the abdominal cavity with warm saline and close the abdomen layer by layer. Vein cross-clamp time and total procedure time were recorded.

#### 2.3.3. Model of sutured vein replacement

##### 2.3.3.1. Donor vein acquisition

After skin preparation and disinfection of the abdomen of donor rats, a 2-cm-long incision was made below the xiphoid process into the abdomen, and the subhepatic vein was exposed after separation of the bowel. After separating the vein from the surrounding tissue space, the vein was exposed throughout, and after blocking the distal and proximal veins, respectively, a vein segment of about 7 mm in length was obtained and washed with heparinized saline and placed in heparinized saline at 4°C for future use (Figure 1E).

##### 2.3.3.2. Donor vein placement

The preparation of recipient rats and the mobilization of veins were the same as before. The vein was temporarily blocked at the distal and proximal ends of the vein with a vascular clamp or silk thread, divided in the middle of the free segment of the vein, and the recipient vein was trimmed according to the length of the donor vein (Figure 1F). At the proximal anastomotic stoma, a needle was sutured at 3 o'clock and 9 o'clock positions of the recipient vein and the donor vein as the traction fixation line, the anterior wall of the vein was anastomosed by the continuous suture method, the posterior wall of the vein was sutured by the continuous suture method after the meridian was turned over; the distal end of the recipient vein was sutured by the same method (Figure 1G), and heparinized saline was injected into the vein to discharge the air in the venous lumen before suturing. The distal and proximal occlusion of the vein was opened in turn (Figure 1H). If there is no active bleeding and the vessel is patent, wash the abdominal cavity with warm saline and close the abdomen layer by layer. Vein cross-clamp time and total procedure time were recorded.

### 2.4. Replacement vessel ultrasound

The rats in the 2 groups were examined by replacement vascular ultrasonography before sacrifice at different time points, and the rats were fixed on the operating table after anesthesia with 1% isoflurane inhalation. They were imaged using a small animal ultrasound imaging system (Vevo2100, Germany) equipped with a transducer (SP6-12) in the frequency range 3–14 MHz, pulse repetition rate set at 2.2–3.3 kHz, a replacement vein found with the inferior renal vein as a marker, two-dimensional and color Doppler images obtained at a frame rate of 16–30 Hz to measure the diameter and blood flow rate.

### 2.5. Histopathological examination

Rats in the two groups underwent laparotomy to obtain replacement vascular samples after vascular ultrasound measurement at different time points, and the rats were sacrificed after exsanguination *via* the vein. The obtained replacement vessels were divided into two segments, one was fixed in 10% formalin for 48 h, dehydrated and then immersed in wax-embedded tissue specimen blocks, sectioned at 4 μm, and stained with HE, CD4 and CD8 immunohistochemistry (Abcam, UK). The mean optical density (MOD) value of the immunohistochemically stained sections was calculated using Image Pro Plus 6.0 software. The MOD value of the immunohistochemical pathological sections of each rat was calculated by randomly selecting three regions under 200X field of view, and the average value was taken as the MOD value of the sections.

### 2.6. Tissue PCR assay

The other segment of replaced vascular tissue was immediately transferred to liquid nitrogen for preservation, and RT-PCR relative quantification of TGF-β1 was performed. We used GAPDH as an internal reference to calculate the relative expression of TGF-β1. Total RNA from specimens was extracted using the TRIzol method (TRIzol, Invitrogen, USA), and cDNA synthesis was performed using the SuperScript First-Strand Synthesis Kit (Thermo, USA). Primers were purchased (Sangon, China) and real-time PCR was performed using the SYBR Green PCR Master Mix (Roche, Switzerland) kit.

Primer Information: GAPDH primer: F: 5′-GGCAAGTTCAACGGCACAG-3′

R: 5′-CGCCAGTAGACTCCACGACA-3′

TGF-β1 primer: F: 5′-ATAGCAACAATTCCTGGCGTTACCTT-3′

R: 5′-CCTGTATTCCGTCTCCTTGGTTCAG-3′.

### 2.7. Statistical methods

All test data were summarized and statistically analyzed using SPSS 19.0 statistical software. Measurement data were expressed as mean ± standard deviation for normal distribution and median (interquartile range) for non-normal distribution. For the comparison of measurement data between the two groups, *t* test was used for normal distribution and rank sum test was used for non-normal distribution. At the same time, the error graph was applied to describe the observation indicators. *P* < 0.05 was routinely taken as statistically significant difference.

## 3. Results

### 3.1. Ultrasonography of operation and replacement vessels in different grouping models

**Figure 2:**
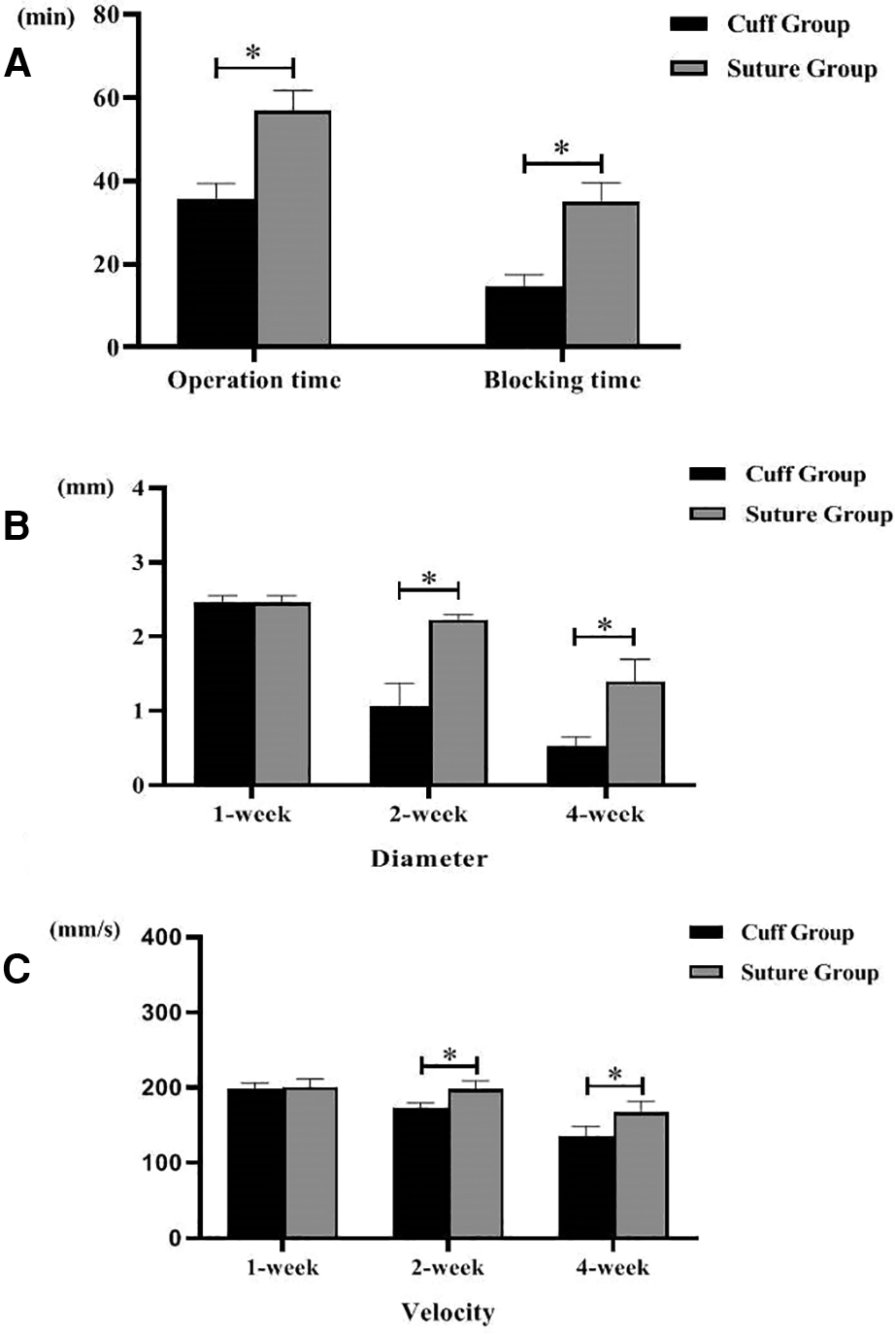
Operation time of two models of vein replacement and postoperative vascular ultrasound (* indicated statistically significant difference between the two groups). (**A**) Operation time and blood flow blocking time of two models. (**B**) Inner diameter of abdominal ultrasound indicating vein at different time points of two models. (**C**) Blood flow velocity of abdominal ultrasound indicating vein at different time points of two models. Error bars represent standard deviation of the mean.

**Figure 3:**
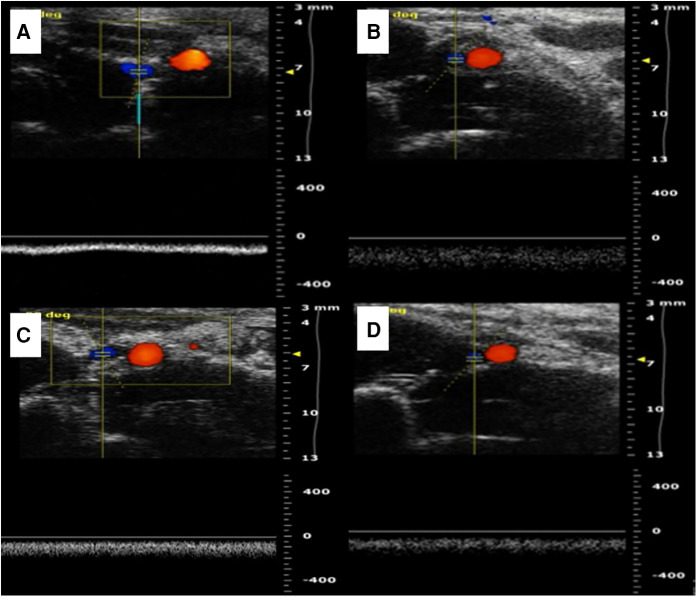
Two kinds of vascular ultrasound in vein replacement model. (**A**) Abdominal ultrasound at 1 week after operation in the cuff group. (**B**) Abdominal ultrasound at 4 weeks after operation in the cuff group. (**C**) Abdominal ultrasound at 1 week after operation in the suture group. (**D**) Abdominal ultrasound at 4 weeks after operation in the suture group.

The total operation time and venous cross-clamp time in the cuff group and suture group were shown in Table 1 and Figure 2A. Operative time and venous cross-clamp time were shorter in the cuff group than in the suture group (*P* < 0.05). The results of Doppler ultrasound at different time points in the cuff group and suture group are shown in Table 2; Figures 2B,C, 3. The results showed that with the prolongation of postoperative survival time, the vascular diameter of the replaced segment in the two groups gradually decreased, and the flow rate gradually slowed down (*P* < 0.05). There was no statistical difference in the vascular diameter and flow rate of the replaced segment in the suture group at 1 week after operation, while the diameter of the suture group was wider than that of the cuff group at 2 and 4 weeks after operation, and the flow rate was faster (*P* < 0.05).

**Table 1:** Surgical conditions of two models of vein replacement.

Item	Cuff group	Suture group
Operation time (min)	35.8 ± 3.6	56.8 ± 4.9*
Cross-clamp time (min)	14.6 ± 2.9	35.1 ± 4.5*

* Indicates *P* < 0.05 compared with rats in cuff group.

**Table 2:** Ultrasonic detection of two kinds of vein replacement models.

Item	Control group	Cuff group			Suture group		
		1-week	2-week	4-week	1-week	2-week	4-week
IVC Diameter (mm)	2.51 ± 0.06	2.45 ± 0.10	1.07 ± 0.31**	0.52 ± 0.13**^,^ ***	2.46 ± 0.09	2.22 ± 0.08*	1.40 ± 0.30*^,^ **^,^ ***
IVC Velocity (mm/s)	201.2 ± 8.5	198.7 ± 7.6	173.3 ± 6.6**	135.2 ± 13.6**^,^ ***	200.7 ± 10.9	197.8 ± 11.4*	167.5 ± 14.3*^,^ **^,^ ***

* Indicates *P* < 0.05 compared with rats in cuff group.

** Indicates *P* < 0.05 compared with rats in 1-week group.

*** Indicates *P* < 0.05 compared with rats in 2-week group.

### 3.2. Histopathology of vascular replacement after operation in different groups

**Figure 4:**
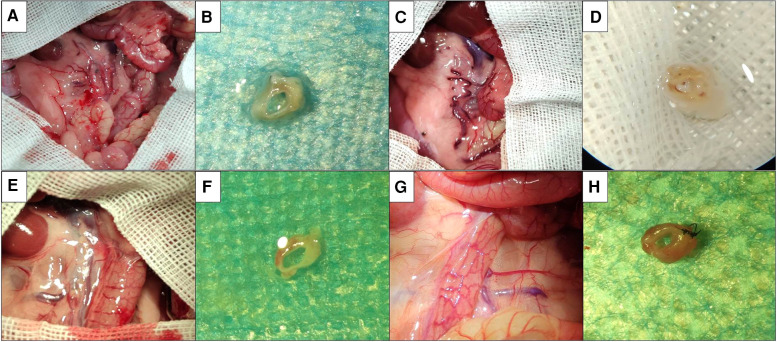
Postoperative gross specimens of two models. (**A**) Abdominal exploration at 1 week after operation in the cuff group showed mild adhesion around the vessels at replacement segment, which showed congestion change of surrounding tissues. (**B**) Gross specimen at 1 week after operation in the cuff group showed that the vessels at replacement segment were unobstructed, and no thrombosis was observed. (**C**) Abdominal exploration at 4 weeks after operation in the cuff group showed that there was significant adhesion around the vessels at replacement segment, which showed formation of a large number of venous collaterals. (**D**) Gross specimen at 4 weeks after operation in the cuff group showed that the vessels at replacement segment were unobstructed, the wall was significantly thickened, which showed fibrous shell. (**E**) Abdominal exploration at 1 week after operation in the suture group showed that there was no significant adhesion around the vessels at replacement segment, which showed formation of venous collaterals. (**F**) Gross specimen at 1 week after operation in the suture group showed that the vessels at replacement segment were unobstructed, which showed good shape. (**G**) Abdominal exploration at 4 weeks after operation in the suture group showed mild adhesion around the vessels at replacement segment, which showed formation of local venous collaterals. (**H**) Gross specimen at 4 weeks after operation in the suture group showed that the vessels were unobstructed, which showed slight thickening of wall.

The gross specimens of the replaced vessels after operation in the cuff suture group are shown in Figure 4. At 1 week after operation, in the cuff group, there were mild adhesions around the vessels in the replaced segment, hyperemic changes in the surrounding tissues, no significant venous collateral formation, and the vessels in the replaced segment were unobstructed. At 4 weeks after operation, a large number of venous collaterals were formed in the replaced segment of the cuff group, cellulose shell was formed outside the wall, and the vascular wall became significantly thicker. One week after operation, no obvious adhesion or venous collateral branch was observed around the vessels in the replaced segment in the suture group. The vessels in the replaced segment were unobstructed, no thrombosis was observed, and no thickening of the wall was observed. At 4 weeks after operation, collateral formation was observed around the replaced segment of vessels in the suture group, with smooth vessels and slightly thickened wall.

**Figure 5:**
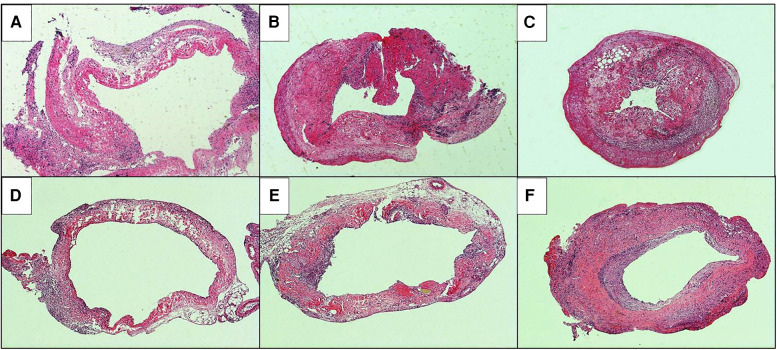
He staining of postoperative replacement vessels in two models of vein replacement (X50). (**A**) At 1 week after operation in the cuff group, a large amount of mononuclear cell infiltration was observed in the adventitial layer of the wall. (**B**) At 2 weeks after operation in the cuff group, mononuclear cell infiltration was observed in the whole wall. (**C**) At 4 weeks after operation in the cuff group, the intimal and muscular layer were significantly thickened, cellulose deposition was observed in the adventitial layer, and mononuclear cell infiltration was observed in the whole wall. (**D**) At 1 week after operation in the suture group, mononuclear cell infiltration was observed in the adventitial layer of the wall. (**E**) At 2 weeks after operation in the suture group, the thickening was observed in the every layer of the vessel wall. (**F**) At 4 weeks after operation in the suture group, the wall was thickened, especially in the intimal and muscular layer, and mononuclear cell infiltration was observed in the every layer of the vessel wall.

The HE staining of the replaced vessels after operation in the sleeve suture group is shown in Figure 5. It can be seen that: 1 week after vein replacement, a large number of mononuclear lymphocyte infiltration was observed in the cuff group and suture group, mainly accumulated in the adventitial layer, and the wall structure was not significantly changed. At 2 weeks after operation, hyperplasia and thickening were observed in the cuff group, and mononuclear cell infiltration was observed in every layer of the vessel wall. In the suture group, mononuclear cell infiltration was also observed in the every layer of the vessel wall, with only mild hyperplasia and no obvious stenosis. At 4 weeks after operation, the intimal layer and muscular layer in the cuff group were significantly thickened, significant fibrous encrustation was observed outside the wall, and mononuclear cell infiltration was observed throughout the wall. In the suture group, mononuclear cell infiltration in the every layer of the vessel wall and thickening of the intimal layer were also observed, but they were milder than those in the cuff group.

### 3.3. Immunohistochemical staining and PCR of replaced vessels after operation in different groups of models

**Figure 6:**
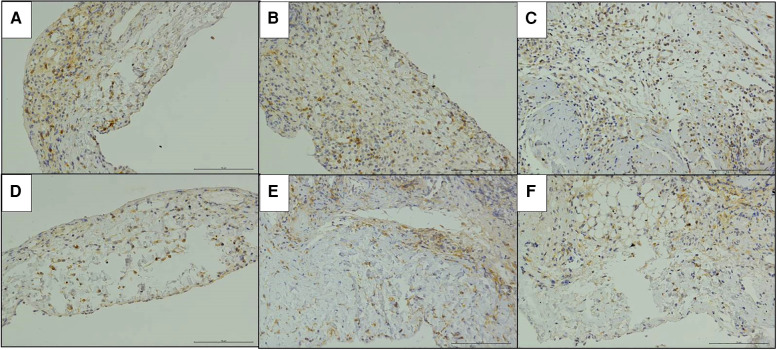
Immunohistochemical staining of CD4 in the replaced vessels after operation in two models of vein replacement (X200). (**A**) At 1 week after operation in the cuff group, patchy yellow-stained CD4+ lymphocytes were observed in the every layer of the vessel wall. (**B**) At 2 weeks after operation in the cuff group, a large amount of yellow-stained CD4+ lymphocyte infiltration was observed in the every layer of the vessel wall. (**C**) At 4 weeks after operation in the cuff group, locally yellow-stained CD4+ lymphocytes were observed in the wall. (**D**) At 1 week after operation in the suture group, yellow-stained CD4+ lymphocytes were scattered in the wall. (**E**) At 2 weeks after operation in the suture group, yellow-stained CD4+ lymphocytes were mainly concentrated in the adventitial layer of the wall. (**F**) At 4 weeks after operation in the suture group, yellow-stained CD4+ lymphocytes were mainly concentrated in the adventitial layer of the wall.

**Figure 7:**
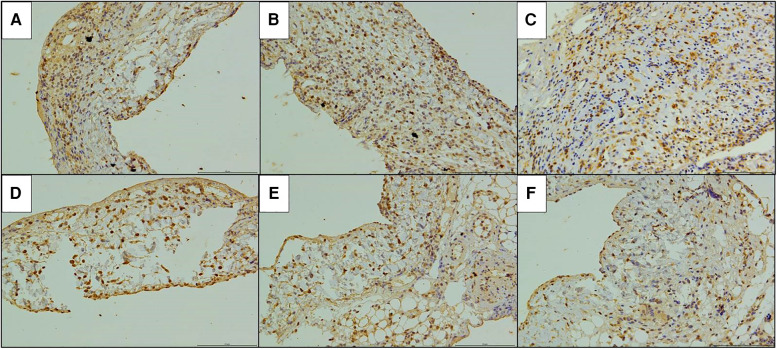
Immunohistochemical staining of CD8 in the replaced vessels after operation in two models of vein replacement (X200). (**A**) At 1 week after operation in the cuff group, patchy yellowish CD8+ lymphocytes were observed on the wall, especially in the adventitial layer. (**B**) At 2 weeks after operation in the cuff group, yellowish CD8+ lymphocytes were observed on the whole layer of the wall. (**C**) At 4 weeks after operation in the cuff group, yellowish CD8+ lymphocytes were observed on the every layer of the vessel wall, with no significant change compared with before. (**D**) At 1 week after operation in the suture group, scattered yellowish CD8+ lymphocytes were observed on the every layer of the vessel wall. (**E**) At 2 weeks after operation in the suture group, yellowish CD8+ lymphocyte infiltration was observed on the every layer of the vessel wall, with no significant change compared with before. (**F**) At 4 weeks after operation in the suture group, yellowish CD8+ lymphocytes were mainly concentrated in the adventitial layer of the wall.

**Figure 8:**
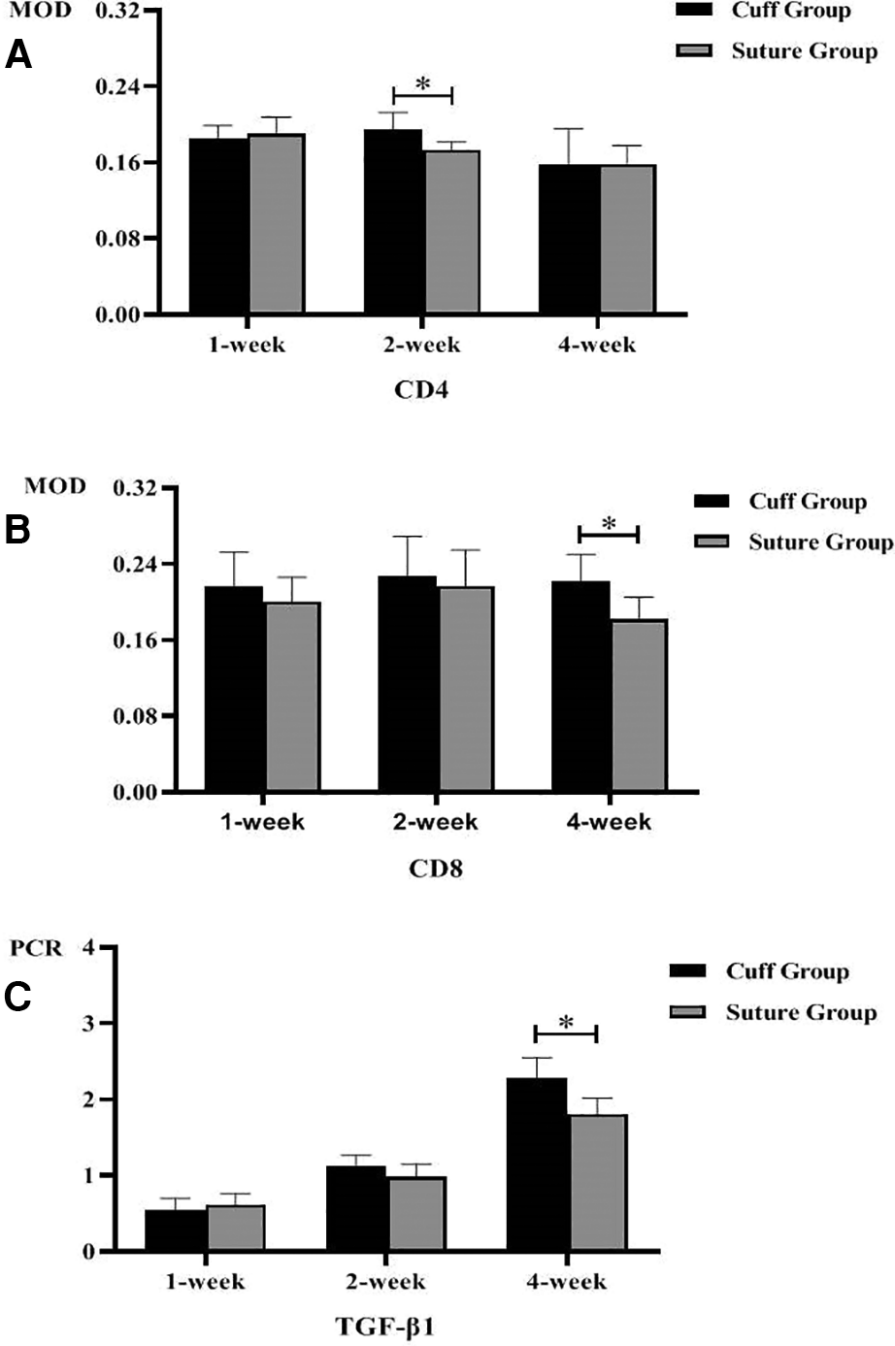
Postoperative MOD value and PCR quantification of replaced vessels in two vein replacement models (* indicates statistically significant difference between the two groups). (**A**) MOD values of CD4 immunohistochemical staining at different time points in the two models. (**B**) MOD values of CD8 immunohistochemical staining at different time points in the two models. (**C**) PCR quantification of TGF-β1 in replaced vessels at different time points in the two models. Error bars represent standard deviation of the mean.

The immunohistochemical staining of CD4 and CD8 in the replaced vessels after surgery in the cuff group and suture group are shown in Figures 6, 7. The MOD value of immunohistochemical staining was shown in Table 3; Figures 8A,B. Thus, after vein replacement, both cuff method and suture method caused CD4+ and CD8+ lymphocyte infiltration, which peaked at about 2 weeks after surgery and then gradually decreased. For CD4+ lymphocytes, the MOD value of suture group was lower than that of cuff group at 2 weeks after operation (*P* < 0.05), and there was no statistical difference at other time points. For CD8+ lymphocytes, the MOD value of suture group was lower than that of cuff group at 4 weeks after operation (*P* < 0.05), and there was no statistical difference at other time points.

**Table 3:** MOD value and PCR quantification of postoperative replacement vessels in two types of vein replacement models.

Item	Control group	Cuff group			Suture group		
		1-week	2-week	4-week	1-week	2-week	4-week
CD4 MOD	0.10 ± 0.02	0.19 ± 0.01	0.20 ± 0.02	0.16 ± 0.04***	0.19 ± 0.02	0.17 ± 0.01*	0.16 ± 0.02***
CD8 MOD	0.10 ± 0.02	0.22 ± 0.04	0.23 ± 0.04	0.22 ± 0.03	0.20 ± 0.03	0.22 ± 0.04	0.18 ± 0.02*^,^ ***
TGF-β1 PCR	0.43 ± 0.12	0.55 ± 0.15	1.12 ± 0.15**	2.28 ± 0.26**^,^***	0.62 ± 0.15	0.98 ± 0.16**	1.80 ± 0.22*^,^ **^,^ ***

* Indicates *P* < 0.05 compared with rats in the cuff group.

** Indicates *P* < 0.05 compared with rats in the 1-week group.

*** Indicates *P* < 0.05 compared with rats in the 2-week group.

The PCR quantitative analysis of TGF-β1 gene in the replaced vessels of the two groups after surgery is shown in Table 3 and Figure 8C. It can be seen that after vein replacement, the content of TGF-β1 gene in both cuff method and suture method showed a gradually increasing trend (*P* < 0.05). At 4 weeks after operation, the content of TGF-β1 gene in suture group was lower than that in cuff group (*P* < 0.05), and there was no statistical difference at other time points.

## 4. Discussion

The successful clinical application of allogenic vessels was first seen in 1949, and Gross et al. (14) first treated aortic constriction with preserved allogenic arteries to reconstruct the left subclavian artery and left pulmonary artery in patients. Then in 1950, Lam (15) and others first successfully applied the allogeneic artery replacement technique to treat patients with thoracic aortic aneurysms. In 1954, Professor DeBakey (16) reported a group of 22 patients with aortic occlusive disease treated with allogeneic arterial replacement technique and described the surgical process of the patients in detail, and only one patient died after surgery due to bleeding from the replaced vessel. Since then, allogenic arteries have been widely used in the treatment of aortic diseases (17–19), but the use of allogenic veins has rarely been reported. Until recent years, with the progress of surgery and the change of the concept of cancer treatment, the surgery combined with venous vascular resection and reconstruction has become an important part of the comprehensive treatment of cancer (20–22), and the surgical methods using allogeneic veins for vascular reconstruction are increasingly reported (23, 24).

Compared with other vascular grafts, the application of allogeneic blood vessels has the advantages of rich sources, no increase in trauma, easy matching of caliber, and good histocompatibility. It is an ideal venous substitute material in clinical work. However, with the increase of clinical application, scholars have found that allogeneic veins also have the risk of vascular stenosis in the long term. Kleive (25) reported the data of 42 patients with pancreatic cancer who underwent portal vein replacement using allogeneic veins in Oslo University Hospital, Norway in the past 10 years. During the follow-up period, 26 patients (61.9%) had vascular stenosis. Kleive also measured HLA-specific antibodies in 13 of these patients, and HLA typing of 10 of the donors revealed that the antibodies were donor specific. Therefore, Kleive believes that the most important cause of vascular stenosis in replacement is local tumor recurrence, but also there is a certain vascular rejection. However, due to the difficulty in clinical sampling, there is no relevant study report on the long-term pathological changes of replaced venous vessels, and the specific pathological changes of long-term thrombosis and anastomotic healing cannot be assessed. Therefore, it is important to study the long-term pathological changes after allogeneic vein replacement in experimental animals to prevent and treat stenosis.

Through literature review, we found that the current small animal model of vascular replacement is the arterial replacement model. Koulack et al. (26), in 1995, reported a replacement model from the thoracic aorta to the abdominal aorta in mice for studying arterial chronic rejection. Zhu et al. (27) subsequently reported a mouse cuff abdominal aortic replacement model for studying arterial rejection. However, the structure of venous wall is different from that of arterial wall, which has fewer fibers and lacks elastic membrane compared with arterial wall, so the long-term pathological changes after replacement may also be different. Zhang et al. (28) investigated the pathological changes of neointima after venous replacement by transplanting the inferior vena cava into the carotid artery in mice. However, after vein replacement into the arterial circulation, the vein gradually expanded under the action of arterial pressure. With the extension of postoperative survival time, the venous wall gradually remodeled and thickened to adapt to the high pressure of arterial circulation. Sometimes the blood formed eddy flow at the junction and fibrin was deposited on the venous folds, which would eventually lead to occlusive thrombosis. Therefore, this model is more suitable for studying venous vascular pathological changes after cardiac bypass or arteriovenous fistulization.

The real small animal vein replacement model is seen in the mouse portal vein replacement model established by Yan et al. (29) through the “double-sleeve” technique. Its study suggests that rejection will occur after allogeneic mouse vein replacement resulting in stenosis, and CTLA4-Ig treatment helps to improve the occurrence of rejection. However, this model has some drawbacks because this technique leaves a polyethylene cuff around the vessel wall in mice. The nonspecific inflammation caused by this cuff *in vivo* may also affect the pathological changes of the transplanted vein, while the authors did not evaluate the vascular fibrosis caused by the inflammatory response, and it cannot be considered that the wall thickening was completely caused by rejection. Therefore, in order to reflect the pathological characteristics after vein replacement, we established a stable rat vein replacement model using both cuff method and suture method.

As to the difficulty of surgical operation, the cuff method has shorter operation time and venous cross-clamp time than the suture method. Moreover, the whole surgical operation process does not require fine suture, and the rats can still survive healthily at 4 weeks after surgery, thus the cuff method seems to be an ideal modeling method. However, our further analysis found that the cuff method was inferior to the suture method in terms of venous collateral formation, diameter of the replaced vessel, and flow rate. Pathological HE staining showed that the cuff method had more significant proliferation of the replaced vessel and intimal layer than the suture method. At 4 weeks after surgery, the intimal layer in the cuff group was significantly thickened. The difference between the two modeling methods is that the polyethylene cuff method will retain polyethylene cuff in the body. We consider that the polyethylene cuff will produce non-specific inflammation in the surrounding tissues, resulting in the thickening of the vascular wall and intimal layer in the cuff, while the fibrinoid package formed by the body exuding around the polyethylene cuff will also deposit in the adventitial layer of the vascular wall to form a fibrous envelope (30, 31), further leading to stenosis. Therefore, we believe that the vein replacement model constructed by the cuff method is greatly affected by the cuff and does not meet the characteristics of clinical actual operation, and is not suitable for the study of long-term pathological changes of the replaced vessels.

After suture, the main pathological changes after vascular replacement were mononuclear lymphocyte infiltration in the vascular wall, which potentially led to the proliferation of blood vessels and intimal layer, without the stenosis caused by obvious thrombosis. We further found by CD4 and CD8 immunohistochemical staining that CD4+ and CD8+ lymphocytes in the wall showed a gradually increasing trend after replacement of blood vessels, peaked at about 2 weeks after surgery, and then gradually decreased. Since the syngeneic background rat was used for vein replacement in this study model, and there was no lymphocyte infiltration caused by rejection, we considered that the main cause of lymphocyte infiltration in the vessel wall was the local inflammatory response caused by surgical suture.

During tissue healing, the inflammatory response is the initiating link in fibrosis (32). To further confirm whether the wall thickening of the replaced vessels was associated with vascular fibrosis, we further measured the TGF-β1 gene content of the vascular tissue. Currently, TGF-β1 is the cytokine known to be most closely related to vascular fibrosis in the body, and can be produced by a variety of cells, especially activated inflammatory cells (33). On the one hand, TGF-β1 can directly stimulate fibroblasts and promote their proliferation, which can be converted into myofibroblasts to vascular fibrosis; on the other hand, it can activate fibroblasts that are otherwise quiescent and promote the formation of collagen matrix. It can also inhibit the decomposition of collagen matrix, resulting in increased continuous accumulation of vascular fibrosis (34, 35). Our results also showed that the expression of TGF-β1 gradually increased after vascular replacement, while the TGF-β1 gene content in the 4-suture group was lower than that in the cuff group (*P* < 0.05), which indicated that the main cause of long-term vascular wall pathological changes may be vascular fibrosis caused by inflammatory response.

This study also has some shortcomings. Because allogeneic vessels used in practical clinical work may have vascular rejection due to different individual genes, which leads to vascular smooth muscle proliferation. However, this study used an isogenic vascular replacement model, it did not involve studies of vascular rejection. In the subsequent further study, we will also further explore the characteristics of rejection in the long-term pathological changes of vascular replacement by vein replacement between allogeneic rats on the basis of this study model.

In summary, the construction of rat vein replacement model by suture method is more suitable for the relevant study of long-term pathological changes after venous vascular replacement than the cuff method. The main pathological change of long-term pathological changes after syngeneic background vein replacement may be vascular fibrosis caused by inflammatory response.

## Data Availability

The original contributions presented in the study are included in the article/Supplementary Material, further inquiries can be directed to the corresponding author/s.
